# Multimodal sleep staging network based on obstructive sleep apnea

**DOI:** 10.3389/fncom.2024.1505746

**Published:** 2024-12-18

**Authors:** Jingxin Fan, Mingfu Zhao, Li Huang, Bin Tang, Lurui Wang, Zhong He, Xiaoling Peng

**Affiliations:** ^1^Central Hospital Affiliated to Chongqing University of Technology (Chongqing Seventh People's Hospital), Chongqing, China; ^2^School of Electrical and Electronic Engineering, Chongqing University of Technology, Chongqing, China; ^3^Chongqing University of Technology Sleep Medicine Collaborative Innovation Laboratory, Chongqing, China; ^4^College of Computer Science and Engineering, Chongqing University of Technology, Chongqing, China

**Keywords:** automatic sleep staging, obstructive sleep apnea, time-frequency representation, multi-scale feature extraction, transition rules

## Abstract

**Background:**

Automatic sleep staging is essential for assessing sleep quality and diagnosing sleep disorders. While previous research has achieved high classification performance, most current sleep staging networks have only been validated in healthy populations, ignoring the impact of Obstructive Sleep Apnea (OSA) on sleep stage classification. In addition, it remains challenging to effectively improve the fine-grained detection of polysomnography (PSG) and capture multi-scale transitions between sleep stages. Therefore, a more widely applicable network is needed for sleep staging.

**Methods:**

This paper introduces MSDC-SSNet, a novel deep learning network for automatic sleep stage classification. MSDC-SSNet transforms two channels of electroencephalogram (EEG) and one channel of electrooculogram (EOG) signals into time-frequency representations to obtain feature sequences at different temporal and frequency scales. An improved Transformer encoder architecture ensures temporal consistency and effectively captures long-term dependencies in EEG and EOG signals. The Multi-Scale Feature Extraction Module (MFEM) employs convolutional layers with varying dilation rates to capture spatial patterns from fine to coarse granularity. It adaptively fuses the weights of features to enhance the robustness of the model. Finally, multiple channel data are integrated to address the heterogeneity between different modalities effectively and alleviate the impact of OSA on sleep stages.

**Results:**

We evaluated MSDC-SSNet on three public datasets and our collection of PSG records of 17 OSA patients. It achieved an accuracy of 80.4% on the OSA dataset. It also outperformed the state-of-the-art methods in terms of accuracy, F1 score, and Cohen's Kappa coefficient on the remaining three datasets.

**Conclusion:**

The MSDC-SSRNet multi-channel sleep staging architecture proposed in this study enhances widespread system applicability by supplementing inter-channel features. It employs multi-scale attention to extract transition rules between sleep stages and effectively integrates multimodal information. Our method address the limitations of single-channel approaches, enhancing interpretability for clinical applications.

## 1 Introduction

Sleep is an essential biological process that is vital for both physical and mental well-being. It significantly influences numerous physiological functions, such as cognitive performance, mood regulation, and immune system function (Weber and Dan, 2016). Numerous studies have shown that the prevalence of sleep disorders has been rising in recent years. A study conducted in Australia found that 41% of women and 42% of men experience sleep issues (McArdle et al., 2020).

Sleep is a dynamic process comprising distinct stages that cycle throughout the night (Berry et al., 2017). The American Academy of Sleep Medicine (AASM) offers standardized guidelines for classifying sleep stages, which are commonly utilized in both clinical practice and research environments. It categorizes sleep into specific stages: Wakefulness (W), Rapid Eye Movement (REM) sleep, and Non-Rapid Eye Movement (NREM) sleep. NREM sleep is further classified into three stages: N1 (light sleep), N2 (moderate sleep), and N3 (deep sleep or slow-wave sleep) (Berry et al., 2012). The AASM sleep stage classification criteria are listed in Table 1.

**Table 1:** Description of different sleep stages.

**Stage**	**Name**	**Description**
N1	Light sleep	Transition from wakefulness to sleep, characterized by slow eye movements, lower muscle activity, and the presence of theta waves in EEG
N2	True sleep	No eye movements, sleep spindles, and K-complexes appear in EEG, higher sleep threshold to disturbances, and cessation of conscious awareness of the external environment
N3	Deep sleep (NREM)	Delta waves predominate the EEG, known as slow-wave sleep (SWS), associated with memory consolidation and restorative processes
R	REM Sleep	Rapid eye movement sleep where dreaming occurs, characterized by rapid eye movements, atonia (loss of muscle tone), and beta waves similar to an awake state in EEG
W	Wakefulness	High frequency and low amplitude EEG patterns, voluntary muscle activity, and the ability to respond to stimuli. Eyes are typically open and moving, and muscletone is present

Sleep stage classification is essential for the diagnosis and treatment of sleep disorders. Polysomnography (PSG) remains the gold standard for diagnosing these conditions and determining sleep stages. Manual sleep staging is resource-intensive, requiring specialized equipment and trained expertise. It is often conducted in a controlled laboratory environment, leading to high costs and limited accessibility (Malhotra et al., 2013). Therefore, automatic sleep staging has become a research hotspot.

OSA refers to partial or complete blockage of the upper airway during sleep, accompanied by discontinuous sleep caused by hypoxia. This disease has a high prevalence and widely affects people around the world, seriously affecting patients' sleep quality and overall health. The apnea-hypopnea index (AHI) of the entire night in PSG determines the current diagnostic criteria for OSA. Standard sleep structure includes stage N1, accounting for 2%–5% of total sleep time (TST); stage N2, accounting for 45%–55%; stage N3, accounting for 15%–25%; and REM, accounting for 20%–25%. OSA patients have a fragmented sleep structure due to frequent awakenings, with increased stage N1 and reduced stage N3 and REM.

Early deep learning models, such as those by Andreotti et al. (2018), utilized convolutional neural networks (CNNs) to extract time-frequency domain features from EEG data. Chambon et al. (2018) further refined this approach by developing a feature extractor using multiple convolutional layers to process various input channels and modalities. To fully exploit the temporal information in Electroencephalogram (EEG) signals, some studies have employed Recurrent Neural Networks (RNNs), including Long Short-Term Memory (LSTM) networks and bi-directional LSTM (BiLSTM) networks. Michielli et al. (2019) proposed a cascaded RNN with two LSTM units . However, basic deep learning networks often encounter limitations due to the short duration of input contexts. Consequently, sequence-to-sequence methods have gained popularity, allowing for the analysis of extended sequences of PSG epochs (Phan et al., 2019b). Tang et al. (2022) developed an end-to-end deep learning model for adaptive sleep staging using ECG signals as input. Amann et al. (2020) converted multichannel raw signals into time-frequency images for a CNN-based model, addressing sleep staging as a joint classification and prediction problem .

Current research on sleep monitoring predominantly utilizes single-channel EEG due to its simplicity, facilitating use in home-based and wearable systems (Toban et al., 2023). However, multi-channel EEG models offer enhanced robustness by incorporating multiple data sources, which proves more effective in clinical settings for accurate diagnosis and treatment of sleep disorders. Specifically, combining electrooculography (EOG) with EEG provides additional valuable information, such as detecting eye movements, which single-channel EEG alone may not reliably capture. These models align closely with expert assessments, improving credibility and interpretability.

To further enhance signal representation, recent advancements advocate for transforming one-dimensional physiological signals into more informative two-dimensional formats like STFT (Guillot and Thorey, 2021), fast Fourier transform (FFT) (Joe and Pyo, 2022), Hilbert-Huang transform (HHT) (Zhang et al., 2020) and wavelet transform (WT) (Kuo et al., 2021), borrowing techniques from image and signal processing domains. Furthermore, similar to the collaborative approaches proposed in computational research across various domains, the application of advanced data filtering and quantization methods can significantly reduce computational complexity, thereby offering potential improvements in the analysis of physiological signals (Babović et al., 2023).

Although these studies have made some progress, some problems still need to be addressed.

**Figure 1:**
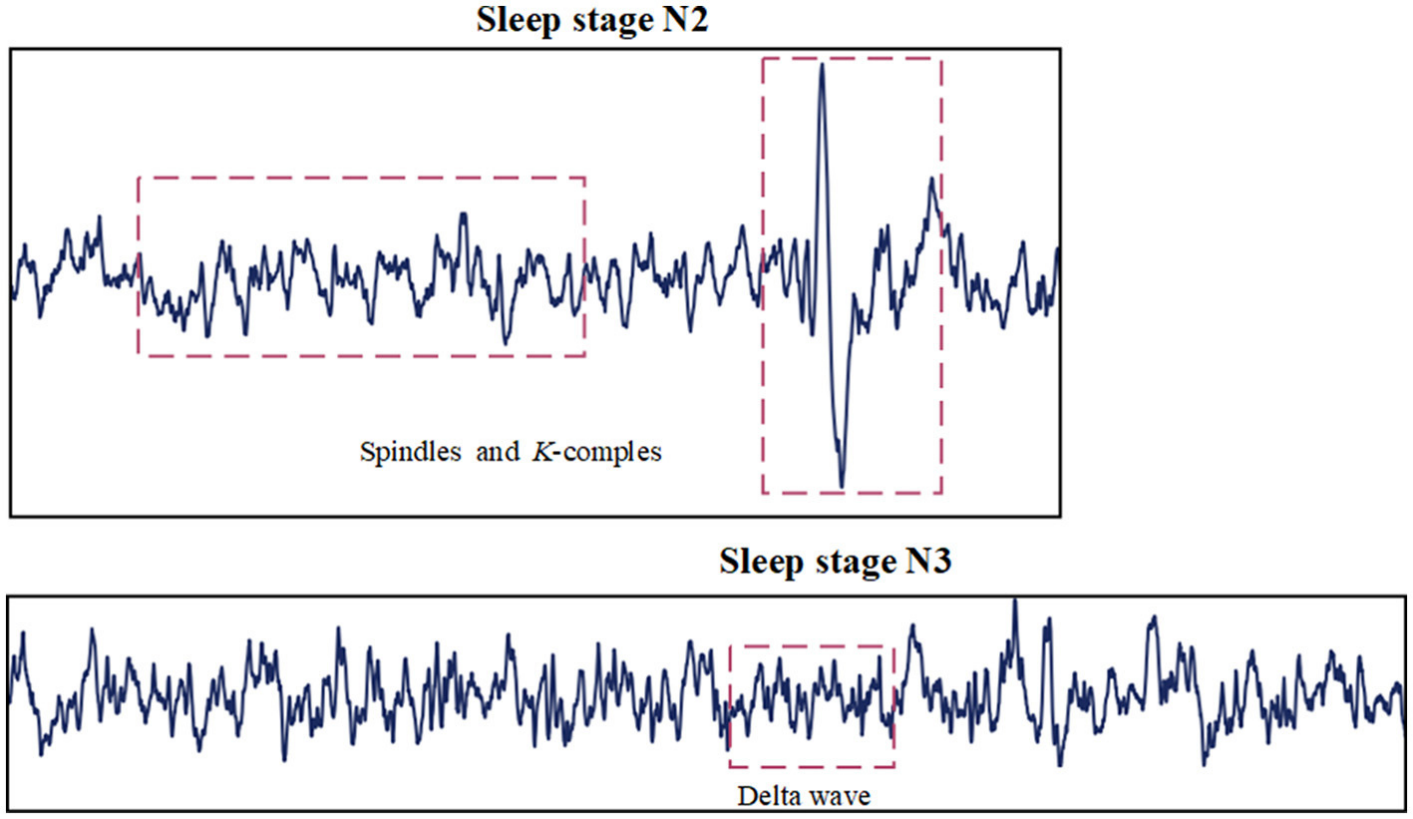
Characteristic waves in sleep stages.

1. The different characteristic waves observed during various sleep stages do not have the same time scale. Characteristic waves refer to specific types of brain activity that are distinctly associated with different sleep stages. These waves vary significantly in frequency, amplitude, and duration, making them crucial for identifying and differentiating sleep stages. As shown in Figure 1, spindle Waves are bursts of oscillatory brain activity that occur predominantly during N2 sleep. They have a frequency range of about 12–16 Hz and typically last about 0.5–3 s. K-complex waves are large waves followed by a slow wave, occurring approximately every 1–1.7 s during N2 sleep. Delta Waves are characteristic of N3 sleep and have a much lower frequency range of about 0.5–4 Hz (Aeschbach and Borbely, 1993). It is worth studying how to extract features across multiple time scales and capture the complex temporal dependencies inherent in sleep signals.

2. Patients with sleep disorders exhibit significant differences in their sleep cycles compared to healthy individuals (Chokroverty, 2010). In healthy individuals, sleep progresses through well-defined cycles of NREM (N1, N2, N3) and REM stages, with relatively stable durations. OSA patients often suffer from more fragmented sleep, frequent awakenings, and transitions between stages. Disorders like insomnia and OSA can disrupt the normal progression through sleep stages, leading to shorter and more frequent REM and NREM cycles. These differences pose several challenges for automated sleep staging. Models trained on data from healthy individuals may generalize poorly to populations with OSA. The atypical waveforms and fragmented nature of disordered sleep make extracting consistent features across different scales challenging. For automated sleep staging to be clinically useful, it must achieve high accuracy across diverse patient populations, including those with OSA patients.

To address the above challenges, we present a Multi-Scale Dilated Convolution Sleep Staging Network (MSDC-SSNet). This network integrates improved Transformer encoders and multi-scale feature extraction. The model utilizes three PSG channels as inputs, including two EEG channels and one electrooculogram (EOG) signal channel. The backbone is an encoder combining causal convolution and a multi-feature extraction module (MFEM). The proposed MFEM effectively extracts different granularity features across different frequency bands. A weighted fusion mechanism dynamically adjusts the weights of frequency features. Using a residual structure also ensures that the model can effectively learn and extract deep spatiotemporal features. Finally, a multi-channel feature fusion module integrates the features, enhancing the overall model's performance and accuracy.

The proposed model offers several significant contributions to the field of automatic sleep staging:

1. A channel-wise Convolutional Temporal Encoder (CCTE) has been proposed. This encoder is designed to independently process and encode time series from multiple channels. We use time feature sequences to learn sleep stage transition rules and reduce the impact of OSA. It integrates causal convolution techniques and introduces a new normalization method called CrossNorm.

2. Multi-Scale Feature Extraction Module (MFEM): The MFEM that utilizes varying receptive fields to extract features across multiple scales. To enhance feature fusion, we have introduced the Multi-Scale Selection Fusion (MSF) method, significantly boosting the representational capacity of extracted features and facilitating a comprehensive analysis of sleep data.

3. Our CSPH dataset is a proprietary collection specially curated from subjects with OSA. It is designed for sleep staging applications, expanding the breadth of applications of the model and promoting the development of sleep staging.

The structure of this paper is organized as follows: Section 2 introduces the automatic sleep staging method based on OSA patients. Section 3 provides a detailed description of the experimental datasets and settings, along with the presentation of experimental results and model stability analysis. Section 4 offers an in-depth discussion of the research findings, focusing on the limitations of the current model and proposing directions for future research. Finally, Section 5 summarizes the key outcomes and contributions of this study.

## 2 Methods

In this section, The structure of the model is proposed. The model combines the advantages of multiscale feature extraction and causal convolution with the robustness of residual networks, aiming at the automatic staging of sleep stages.

### 2.1 Overview of the model

**Figure 2:**
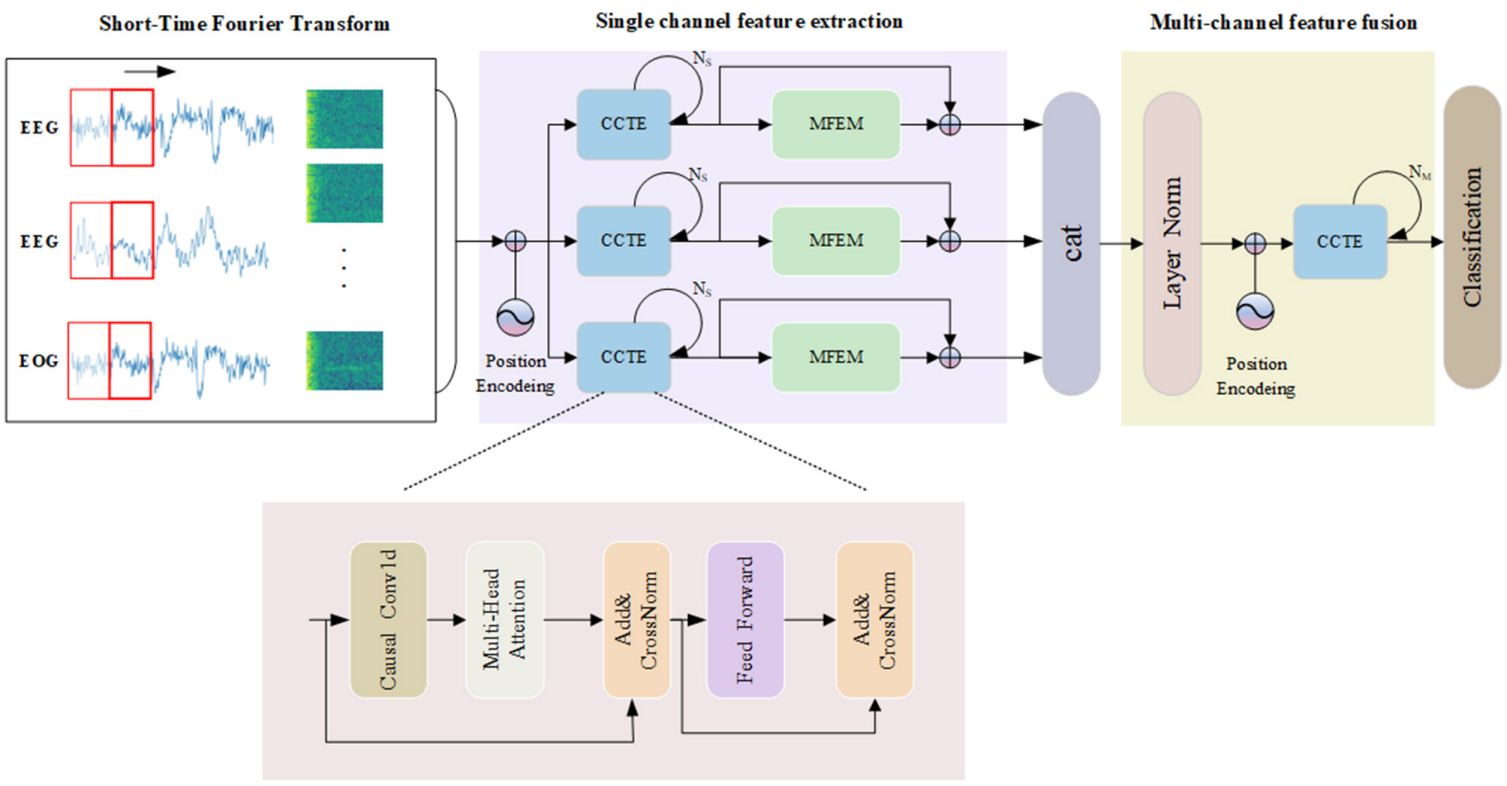
The overall framework of the MSDC-SSRNet model used for automatic sleep stage classification, which contains the CCTE structure.

Figure 2 presents the architecture of our model, which is organized into three key segments: transforming time-frequency data into images, extracting features from individual channels, and integrating and classifying signals from multiple channels. First, the original signal is converted into a time-frequency image by STFT, and the CCTE module is utilized to extract long-range dependent features. Second, the MFEM module adaptively selects important features and fuses the inter-dependencies between single-channel features, which helps to improve the classification performance. By employing residual connections, we fuse multi-scale information with long-range dependency information. Ultimately, channel fusion is utilized to further address the heterogeneity of multimodal physiological signals. In the next section, each module is explained in detail.

### 2.2 Time-frequency image conversion

The model receives input in the form of time-frequency images, designed to preserve specific wave and frequency components of the original signal. According to the AASM scoring guidelines, different physiological electrical signals contribute differently to sleep staging. EEG, EOG, EMG, and other metrics serve as foundational elements in sleep classification. From PSG files, two channels of EEG and one channel of EOG are extracted. Each channel's raw signals undergo STFT and logarithmic scale transformations to generate time-frequency images, which serve as inputs to the model.

Different PSG channels variably contribute to sleep stage classification due to the complex nature of sleep signals and the specific characteristics of each stage. EEG signals are crucial in classifying N2 and N3 stages, marked by distinct waveforms such as sleep spindles, K-complexes, and high-amplitude delta waves. These features are strong indicators of deeper sleep stages and are more readily identifiable in EEG recordings. EOG Signals are more effective in distinguishing REM sleep from N1 sleep. REM sleep is characterized by rapid eye movements, which EOG distinctly captures, whereas EEG signals in REM and N1 stages can appear similar, making EOG a critical component for accurate classification. Therefore, two EEG channels and one EOG channel were extracted from the PSG files.

### 2.3 Channel-wise convolutional temporal encoder

In processing EEG data, a model's comprehensive interpretation of the temporal directionality inherent within time series data is crucial. Traditional Transformer models, due to the characteristics of their self-attention mechanisms, cannot inherently handle the temporal order of time series data. The Channel-wise Convolutional Temporal Encoder (CCTE) integrates causal convolution layers, which inherently maintain the correctness of temporal sequencing by ensuring that the model processes a current data using only the preceding data, thereby effectively preventing the leakage of future information. Furthermore, drawing inspiration from the work of Tang et al. (2021), we innovatively applied the CrossNorm normalization method to the CCTE architecture to enhance the model's performance in processing multi-channel physiological signals. This enables the model to process large-scale time series data more efficiently while maintaining robust performance.

**Figure 3:**
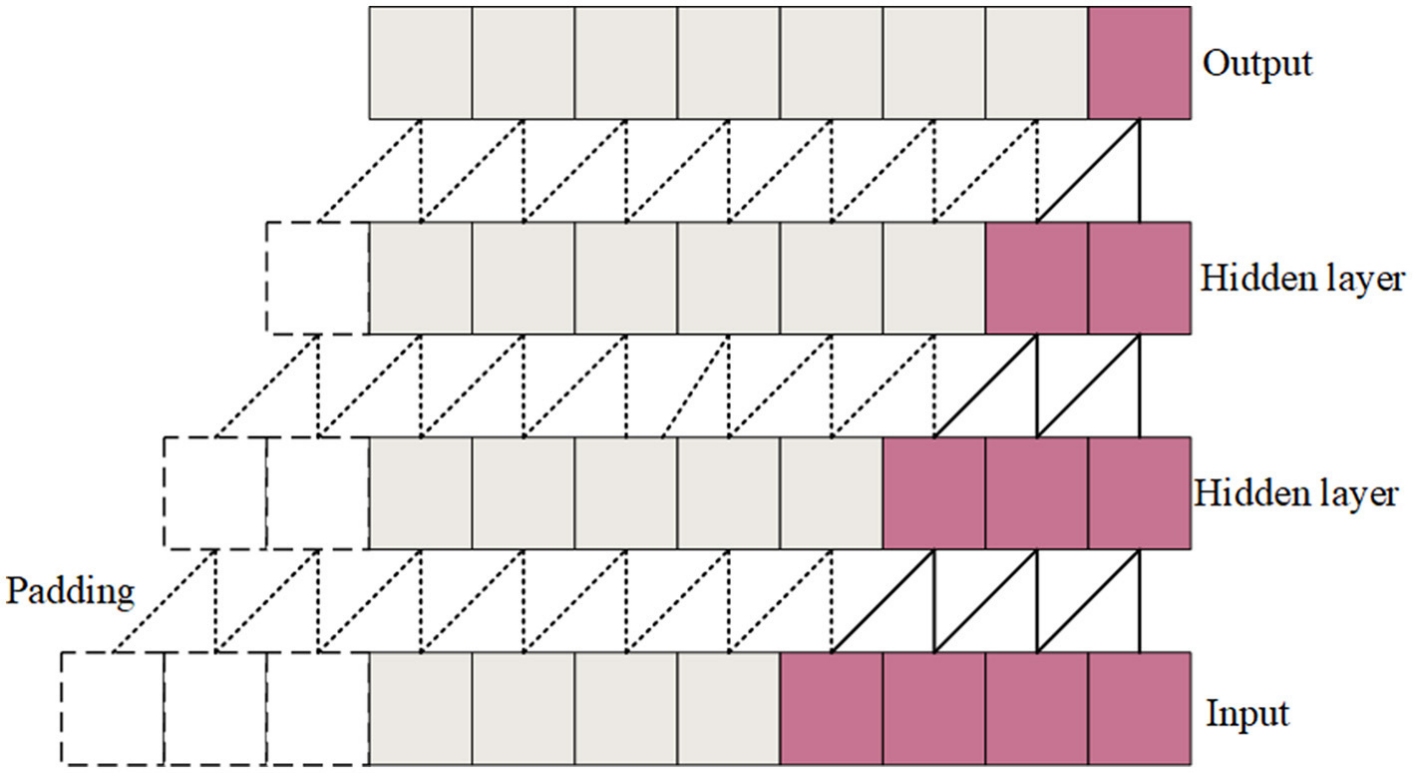
Causal convolution structural diagram.

Causal convolutions are convolutional operations where each output at a specific time step depends only on the current and previous time steps, not future time steps. The causal convolution structure is shown in Figure 3. During the convolution operation, each element of the convolution kernel multiplies only with the current and previous elements of the input data. Padding is employed to ensure that the output sequence is temporally aligned with the input sequence. This property is crucial for maintaining the temporal order of the data. The Channel-wise Convolutional Temporal Encoder (CCTE) is designed to capture time-dependent features in time-frequency images. Traditionally, an Encoder-Decoder module is used for reconstruction tasks. However, since this paper focuses on classification, only the encoder is employed. The core components of the CCTE encoder include the multi-head attention layer, the position feed-forward network, and the normalization layer. By preserving the temporal order, causal convolutions ensure that the model respects the sequence of events in the EEG signal, essential for accurately identifying transitions between sleep stages. The structure of the CCTE module is shown in Figure 2.

#### 2.3.1 Multi-head attention

**Figure 4:**
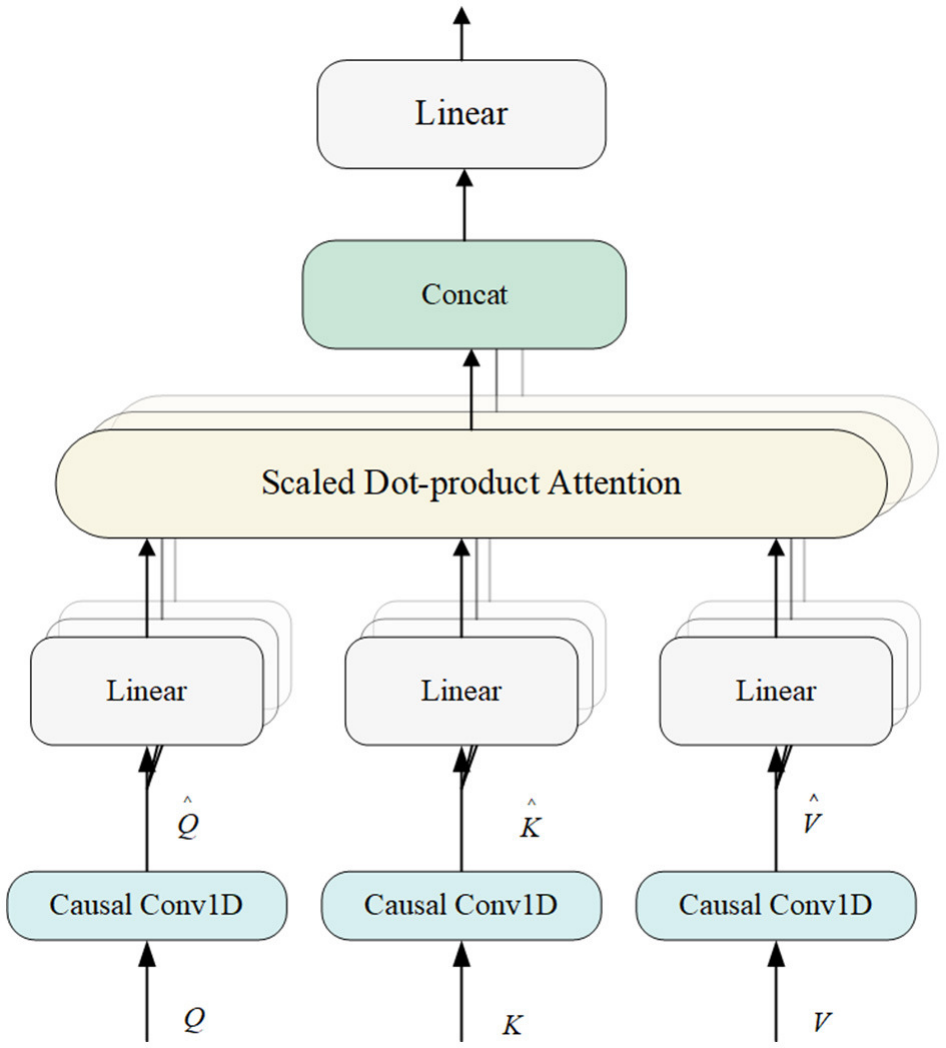
Structure of multi-head attention module.

Multi-head Attention (MHA) is an effective time series data model method (Devlin et al., 2018). The Transformer model has gained popularity due to its successful handling of long-distance dependencies in sequential data. MHA employs multiple attention heads, each of which can learn information from different subspaces of the input data. This allows the model to capture a wide range of features. While a single attention head might focus on the most prominent features, multiple heads can also capture subtle details that might be missed otherwise. For sleep staging, the model can better interpretation the complex and varied patterns present in EEG signals. This parallel processing increases the model's ability to capture diverse information, improving classification efficiency and effectiveness. The structure of MHA is shown in Figure 4.

The model's use of MHA combined with causal convolution ensures that only previous inputs are relied upon when computing the current output, thus maintaining the temporal order of the sequence and enhancing the model's ability to capture temporal dependencies. The combination of position encoding provides explicit information about the position of elements in the sequence, allowing the model to obtain both explicit information about the position (via position encoding) and implicit temporal dependencies (via causal convolution), which is an effective strategy for dealing with features from different frequency domains.

The MHA module begins by accepting the output from the previous module, represented as $X=\left\{x_{1},\ldots ,x_{N}\right\}\in {\mathbb{R}}^{M\times N}$, where *M* is the total number of features and *n* is the length of *x*_*i*_ for 1 ≤ *i* ≤ *M*. MHA utilizes three copies of *X*, referred to as *Q*, *K*, and *V*. Initially, causal convolution is applied to generate $\hat{Q}$, $\hat{K}$, and $\hat{V}$. The output from the causal convolution is then processed through the attention mechanism.


(1) $$\begin{array}{l} \text{Attention}\left(\hat{Q},\hat{K},\hat{V}\right)=\text{softmax}\left(\frac{\hat{Q}{\hat{K}}^{T}}{\sqrt{F}}\right)V_{i} \end{array}$$

Each matrix is partitioned into *H* subspaces to support a multi-head attention (MHA) implementation, where the heads of each attention result are concatenated to form the final output.


(2) $$\begin{array}{l} MHA\left(\hat{Q},\hat{K},\hat{V}\right)=\text{Concat}\left(A^{1},\ldots ,A^{H}\right)\in {\mathbb{R}}^{M\times n} \end{array}$$

#### 2.3.2 Add and CrossNorm

The final features extracted by CCTE are generated by stacking two identical networks. The output from the previous layer is input into the next layer through a residual connection, followed by layer normalization. We utilize CrossNorm for normalization. Unlike traditional normalization methods, CrossNorm improves the model's adaptability to changes within the data by dynamically replacing the mean and standard deviation from different channels. The introduction of CrossNorm significantly improves the model's ability to capture the characteristics of different sleep stages when analyzing multi-physiological signals and time series data.


(3) $$\begin{array}{l} \frac{B-N_{b}}{M_{a}}+\frac{N_{a}}{M_{b}} \end{array}$$

(4) $$\begin{array}{l} \frac{A-N_{a}}{M_{b}}+\frac{N_{b}}{M_{a}} \end{array}$$

The formula exchanges the standard deviation *M*_*a*_ and mean *N*_*a*_ of channel A with the standard deviation *M*_*b*_ and mean *N*_*b*_ of channel B. Thus, A and B are cross-normalized. Each instance or channel has a unique style. During training, CrossNorm is applied for efficient style enhancement, expanding the training distribution to improve the generalization robustness under distribution changes. Effectively suppresses the impact of frequent transitions in sleep stages caused by OSA.

### 2.4 Feature fusion

The multichannel feature fusion module integrates feature maps from three distinct channels, concatenating them along the column axis to form a comprehensive composite feature map. This approach maximizes the preservation of each channel's unique characteristics. Since different PSG channels contain a lot of similar information, a dropout layer is introduced at the output of multiple channels to reduce the risk of overfitting of the model. Additionally, layer normalization ensures consistent data standardization throughout training, promoting accelerated convergence in the training process.

Multiple Channel-wise Convolutional Temporal Encoders (CCTEs) are employed to capture the joint features extracted from the integrated multichannel feature map. Before inputting the feature map into the encoders, positional encoding is applied to enhance the model's ability to recognize the input sequence's positional context.

### 2.5 Multi-scale feature extraction module

In the context of PSG signals, features across various scales play distinct roles in elucidating sleep states. Drawing inspiration from the concept of feature pyramids (Lin et al., 2017), we propose a novel module named the Multi-Scale Feature Extraction Module (MFEM) to capture multi-scale features effectively.

In the MFEM, convolutional layers with varying dilation rates enable the network to process information across local and broader spatial extents. This capability facilitates the detection of subtle physiological signals that indicate transitions between sleep stages, thereby enhancing accuracy by capturing detailed signal complexities and increasing robustness against noise and variability in signal characteristics. Additionally, to optimize multi-scale pattern recognition, the module balances and integrates features from different scales to maximize their relevance to specific sleep stages.

**Figure 5:**
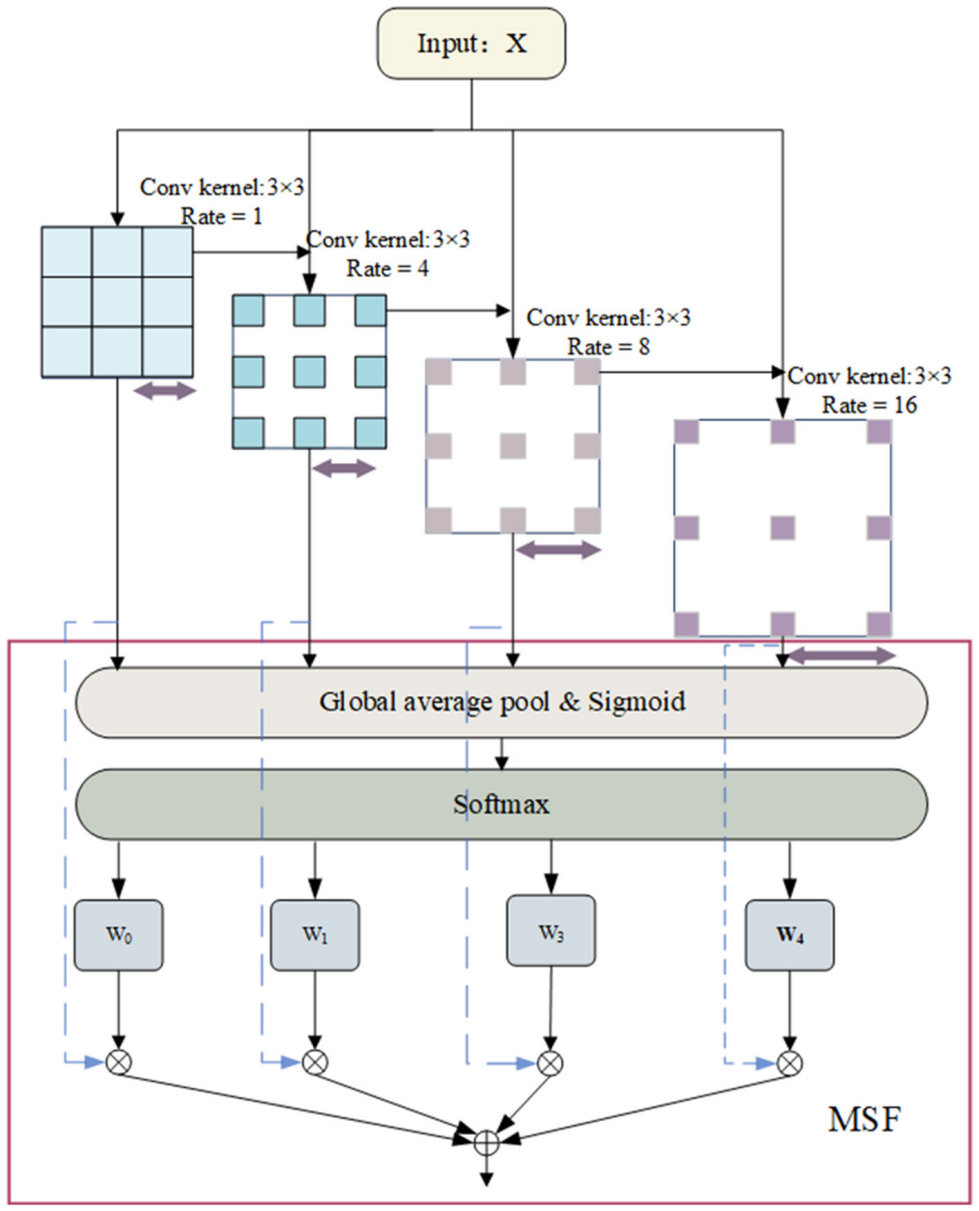
Structure of multi-scale feature extraction module.

Specifically, the MFEM module employs four 3 × 3 atrous convolutions with different dilation rates to convolve the input, producing four sets of feature maps. These feature maps represent information within different frequency ranges. Subsequently, these feature maps are fused to obtain a weighted representation across multiple scales. The operation of the Multi-Scale Feature Extraction Module is illustrated as shown in Figure 5.

In the first step, for an input *x*, convolve it using four 3 × 3 convolutional kernels with dilation rates of [1, 4, 8, 16] to produce four feature maps at different frequencies, denoted as *X*_1_, *X*_2_, *X*_3_, and *X*_4_. Using convolutional kernels with lower dilation rates allows for capturing fine details and local features within the data. These typically correspond to high-frequency variations, such as transient spikes or rapid electroencephalographic (EEG) signal fluctuations. Conversely, employing convolutional kernels with more significant dilation rates enables the detection of broader spatial regions, thus capturing coarse-grained, global, or low-frequency features in the signal.


(5) $$\begin{array}{l} X_{\text{i}}=\left\{ \begin{array}{ll} \text{Cov3}\times 3_{\text{rate}=1}\left(X\right) & \;i=1\\ \text{Cov3}\times 3_{\text{rate}=2\left(i-1\right)}\left(X+X_{i-1}\right) & \;1<i\leq n \end{array}\right. \end{array}$$

In the second step, perform global average pooling (GAP) along the temporal dimension on *X*_1_, *X*_2_, *X*_3_, and *X*_4_ to obtain global feature representations *X*_1_, *X*_2_, *X*_3_, and *X*_4_.

In our experiments, we set the number of atrous convolutions to 4. Different expansion rates enable the network to capture a broader range of spatial contextual information. This architectural design effectively enhances information extraction across various temporal and frequency dimensions by widening its scope while maintaining depth. Following feature extraction, we employ a novel fusion technique known as Multi-Scale Fusion (MSF) to integrate features *Y*_i_ from different scales. The model can adaptively emphasize more significant frequency features and suppress less pertinent information by computing global weights for feature maps at different scales and performing a weighted fusion. Ultimately, the input features X are summed with these fused features. As depicted in Figure 5, the process begins with Global Average Pooling (GAP) being applied to multi-scale features to obtain their mean channel-wise weights (Lin et al., 2013). A Sigmoid activation function is applied to transform these weights into values between 0 and 1. Subsequently, a softmax operation normalizes the average channel weights across multi-scale features to their corresponding positions. Ultimately, the normalized weights multiply their respective features, aggregating these elements to enhance multi-scale features. Due to the combination of convolutional and attentional mechanisms, the MFEM excels in analyzing EEG time-frequency data, effectively extracting and utilizing multi-scale and multi-frequency features of the signal. This capability greatly improves the model's performance in sleep staging, facilitating more precise evaluations of sleep quality through a thorough analysis of EEG characteristics.

## 3 Results

Our analysis employs four distinct datasets to assess the model's performance: Sleep-EDF-20, Sleep-EDF-78, Sleep Heart HealthStudy (SHHS) and Chongqing Seventh People's Hospital (CSPH) data. These datasets are detailed in Table 2.

**Table 2:** Detailed information on the four datasets (each sample is a 30-s calendar element).

**Dataset**	**Subject**	**Sampling rate**	**W**	**N1**	**N2**	**N3**	**REM**	**Total samples**
Sleep-EDF-20	20	100 HZ	9,118 21.1%	2,804 6.50%	17,799 41.30%	5,703 13.20%	7,717 17.90%	43,141
Sleep-EDF-78	78	100 HZ	66,822 34.00%	21,522 11.00%	69,132 35.20%	13,039 6.60%	25,835 13.20%	196,350
SHHS	329	125 HZ	43,619 14.3%	10,304 3.20%	142,125 43.70%	60,153 18.50%	65,953 20.30%	324,854
CSPH	17	512 HZ	4,077 21.9%	2,920 15.7%	8,273 44.4%	1,380 7.4%	1,983 10.7%	13,670

The Sleep-EDF-20 dataset, obtained from PhysioBank (Goldberger et al., 2000), was utilized in two distinct research studies. The initial study, known as the Sleep Cassette (SC) study, involved 20 participants aged 25–34, focusing on exploring the connection between age and sleep patterns in healthy individuals. The second study focused on the effects of temazepam on the sleep patterns of 22 Caucasian males and females who were not taking any medication (Phan et al., 2019b,a; Sokolovsky et al., 2019; Li et al., 2021). Our work utilizes the SC subset. The Sleep-EDF-20 dataset consists of polysomnographic (PSG) recordings, which include multiple physiological signals collected during participants' sleep, such as EEG, EOG, EMG, and others. In the study, two EEG channels and one EOG channel have a sampling frequency of 100Hz. During the experiments, We used Fpz-Cz, Pz-Oz and ROC-LOC (EOG) as the input of the model.

Sleep-EDF-78 is an extension of the Sleep-EDF dataset (Goldberger et al., 2000; Kemp et al., 2000), also sourced from PhysioBank. The age range of the participants has been expanded to include individuals aged 25–101 years, encompassing a total of 78 subjects. To ensure the consistency of the experiment, the same channels as Sleep-EDF-20 were used for analysis.

The SHHS is established to examine how sleep-disordered breathing influences cardiovascular health and a range of other outcomes. It includes full-night PSG recordings involving comprehensive sleep studies with multiple physiological signals. The SHHS Visit 1 comprises 6,441 participants, all aged 40 and above. SHHS Visit 2 consists of 3,295 participants, all from Visit 1. Based on previous studies (Zhao et al., 2022; Eldele et al., 2021), we selected 329 participants with normal sleep rhythms for experimentation, using the C4-A1, C3-A2 and LOC EOG channels as model inputs.

CSPH: This dataset, provided by the Department of Sleep and Psychosomatic Medicine of Chongqing Seventh People's Hospital, China, comprises PSG recordings from 17 subjects aged 20–60 years with OSA. The recordings were sampled at 512 Hz, and each subject underwent manual sleep stage scoring by three sleep specialists following AASM criteria. The PSG recording channels included F4-A1, C4-A1, O2-A1, F3-A2, C3-A2, O1-A2, along with electrooculograms EOGL and EOGR. For analysis, inputs were derived from F4-A1, F3-A2, and EOGL channels. All three datasets employ the AASM sleep scoring standards.

These datasets cover a broad range of subjects, including healthy individuals, those with sleep disorders, and participants across a wide age range, from young adults to older individuals. They provide a diverse set of conditions and scenarios, making the model robust across various sleep patterns.

### 3.1 Experimental setup

A 30-second segment (epoch) of PSG data was sampled for the analysis. The Short-Time Fourier Transform (STFT) is applied using a 2-s Hamming window with 50% overlap. The FFT is computed with 256 points, providing a frequency resolution adequate for sleep analysis. The resultant spectrum is then log-scaled. The resulting time-frequency representation, denoted as *S*∈ℝ^*T*×*F*^, consists of *F* = 128 frequency bins and *T* = 29 time points. This normalized representation is subsequently utilized as the model's input.

In our CCTE encoder, the Multi-Head Attention (MHA) utilizes eight heads and 150 feedforward hidden units. The CCTE modules at the model input and output use different numbers of encoders, *N*_*s*_ = 8,*N*_*m*_ = 4 respectively. Throughout the entire CCTE model, including the self-attention layers, feedforward layers, and fully connected (FC) layers, a uniform dropout rate of 0.1 is applied.

To address the issue of a limited number of subjects, we employed K-fold cross-validation to train the model on four datasets. The values of K for the Sleep-EDF-20, Sleep-EDF-78, SHHS, and CSPH datasets were set to 20, 10, 10, and 10, respectively. Although some datasets, such as Sleep-EDF-20 and CSPH, have a smaller sample size, K-fold cross-validation effectively improved the model's generalization ability and reduced the risk of overfitting through repeated training and validation. Meanwhile, the larger dataset (SHHS) further enhanced the model's stability and robustness, ensuring effective performance across all datasets.The training objective utilized was the cross-entropy loss function, which is commonly used in classification tasks. We used the AdamW (Loshchilov and Hutter, 2017) optimizer, which is more effective in handling weight decay, with a learning rate set to 5 × 10^−5^.Additionally, during the model training process, we employed early stopping, which involves halting training when the performance on the validation set no longer improves, in order to prevent the model from overfitting to the training set.

### 3.2 Evaluation metrics

The model's overall performance is assessed using three key metrics: accuracy (*ACC*), macro-average F1 score (*MF1*), and Cohen's Kappa (κ). The *MF1* is calculated as the arithmetic mean of the F1 scores for the five sleep stages. Precision (*Pre*), recall (*Rec*), and F1-score (*F1*) are used to assess each class individually. The overall accuracy (*ACC*) and macro-average F1 score (*MF1*) are defined as follows:


(6) $$\begin{array}{l} \text{MF1}=\frac{\sum_{c=1}^{C}F1_{c}}{C} \end{array}$$

(7) $$\begin{array}{l} \text{ACC}=\frac{\sum_{c=1}^{C}TP_{c}}{M} \end{array}$$

For each class *c*, the within-class F1-score is denoted as *F*1_*c*_. There are *C* distinct sleep stage categories. For each category *c*, *TP*_*c*_ represents the true positives of that category. Additionally, *M* represents the total number of EEG epochs.

### 3.3 Experimental scoring results

Experimental scoring results are presented in Table 3, using confusion matrices to display the performance of the model. In these matrices, rows represent the actual results, while columns represent the predicted results. Bold numbers within the matrices highlight epochs correctly classified by the model. Evaluation metrics for each category are provided on the right side of the tables, with optimal values emphasized in bold.

**Table 3:** Confusion matrices for different datasets.

**Predicted**						**Per-class metrics**		
**Dataset**	**W**	**N1**	**N2**	**N3**	**REM**	**PR**	**PE**	**F1**
	Sleep-EDF-20	**8,186**	283	75	31	109	94.7	94.2	94.4
		293	**1328**	477	12	561	63.4	49.7	55.7
		89	275	**15,361**	576	650	91.3	90.6	90.9
		9	1	503	**4,915**	3	88.7	90.4	89.6
64		207	408	4	**6,666**	83.4	90.7	86.9
Sleep-EDF-78	**61,287**	2,366	446	75	349	94.3	94.9	94.7
	2,910	**11,441**	6,342	61	2,132	63.2	49.9	55.9
	3,799	2,766	**72,533**	365	2,096	86.0	89.7	87.8
	31	7	2,195	**15,451**	2	82.6	87.3	84.9
	328	1,503	2,763	32	**26,450**	85.2	85.1	85.2
SHHS	**42,853**	1,030	1,317	171	848	93.4	92.7	93.0
	1,488	**5,547**	260	113	2,896	55.9	53.8	54.8
	517	36	**25,041**	4,264	267	81.5	83.1	82.3
	45	1,008	4,072	**50,673**	4,355	86.1	84.2	85.2
	998	2,296	4	3,633	**59,022**	87.5	89.5	88.5
CSPH	**3,267**	254	124	6	55	85.6	88.2	86.9
	342	**1,510**	619	18	165	62.1	56.9	59.4
	130	515	**6,347**	234	295	85.4	84.4	84.9
	5	5	141	**1,104**	0	81.0	88.0	84.3
	73	147	197	1	**1,385**	72.9	76.8	74.8

Bold numbers in the table represent the correct sample counts for each category.

According to the evaluation results of three healthy population datasets, the accuracy of the Wake stage can reach more than 93%. The indicators of the N1 stage are lower than those of the W, N2, N3, REM, and other stages, which may be related to the small number of occurrences of the N1 stage in the data set. Misclassifications frequently occur among the sleep stages, with the W stage often being mistaken for the N1, N2, and REM stages. Similarly, the N1 stage is commonly misclassified as W, N2, or REM, while the REM stage is often confused with N1 and N2. Additionally, the N3 stage is primarily confused with the N2 stage.

For OSA patients in CSPH, the accuracy for the N1 stage can reach 62.1%, while the accuracies for the W stage and N2 stage exceed 85%. However, the model's overall performance is generally lower than that of healthy subjects, reflecting the interference of OSA on sleep staging.

**Figure 6:**
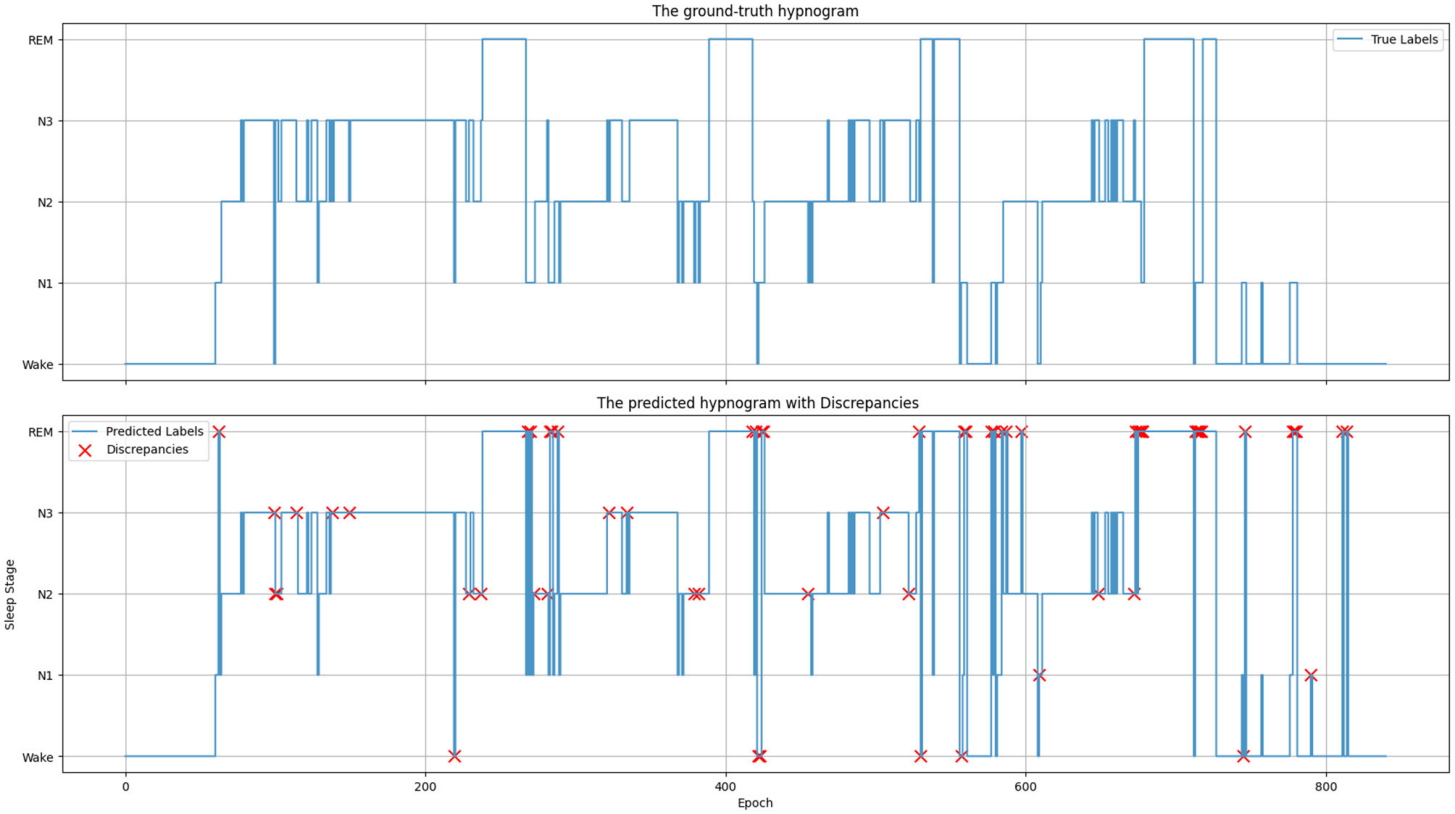
Ground-truth and predicted hypnograms of subject SC4001E0 in the sleep-EDF-20 dataset.

Figure 6 depicts the ground truth and predicted hypnograms for subject SC4001E0 from the Sleep-EDF-20 dataset to further illustrate the findings. The close resemblance between the predicted and true hypnograms demonstrates the model's accuracy. However, the transition into the REM stage exhibits a higher error rate. This primarily arises from the increased variability in EEG signals during transitions and the substantial similarity between mixed-frequency EEGs.

**Figure 7:**
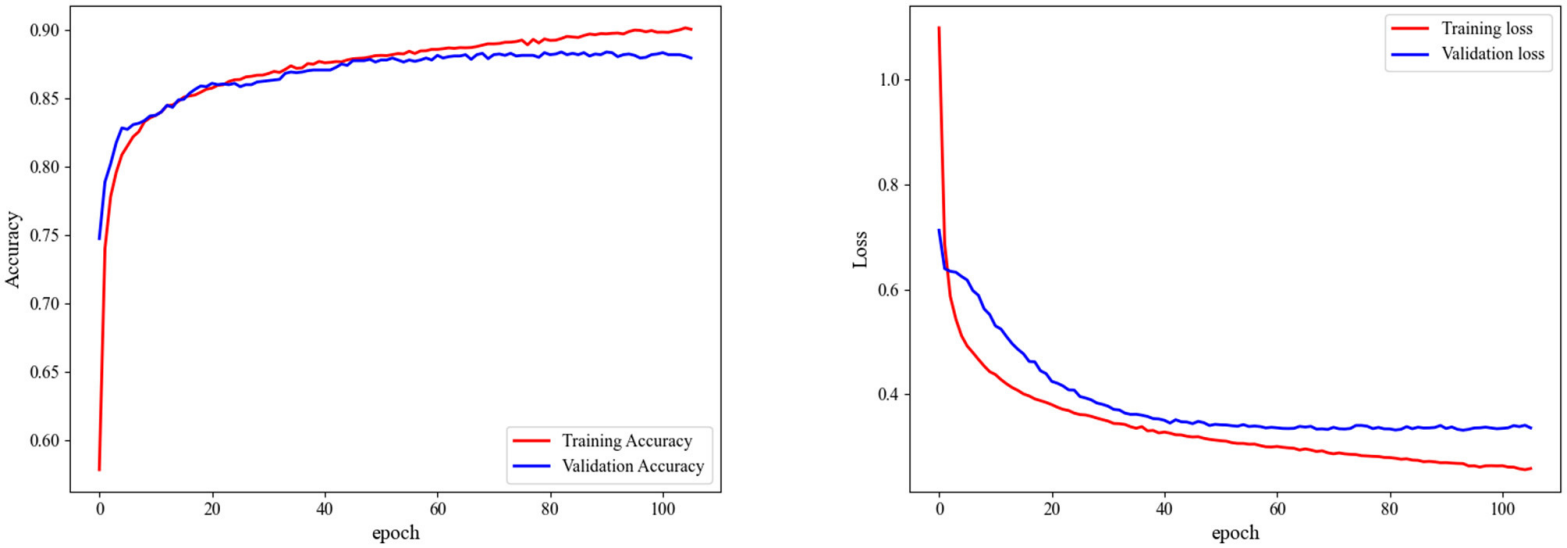
Accuracy and loss during training on fold 6 in the sleep-EDF-20 dataset.

In Figure 7, we present the accuracy and loss curves during the training process for the Sleep-EDF-20 dataset, explicitly focusing on fold 6 selected at random. It is observed that our model can rapidly converge and stabilize at a fixed value soon after training initiation. The accuracy continually improves, and the loss consistently decreases. Similarly, validation sets accuracy and loss values to stabilize, underscoring the model's efficacy in mitigating overfitting.

### 3.4 Performance comparison

We compared our MSDC-SSRNet with previous state-of-the-art methods, evaluating overall accuracy, Cohen's kappa (κ), and MF1 across four datasets, along with the F1-score for each sleep stage. The results are presented in Table 4. Our MSDC-SSRNet exhibits significantly better performance than other models based on the experimental outcomes. On the Sleep-EDF-20 dataset, our model showed improvements of 0.9% in accuracy, 1.2% in kappa, and 2.1% in MF1 over the SleepViTransformer (Peng et al., 2023). It also outperformed the transformer-based multichannel model MultiChannelSleepNet (Dai et al., 2023), with increases of 2.2% in accuracy, 3.0% in kappa, and 3.3% in MF1.

**Table 4:** Comparison of sleep staging performance with previous studies across four experimental datasets.

**Dataset**	**System**	**Over metrics**			**Per-class F1-score**				
		**ACC**	**Kappa**	**MF1**	**W**	**N1**	**N2**	**N3**	**REM**
Sleep-EDF-20	MSDC-SSRNet	**88.7**	**84.6**	**83.5**	**94.5**	**55.7**	**90.9**	**89.6**	86.9
	SleepViTransformer (Peng et al., 2023)	87.8	83.4	81.5	93.8	48.4	89.2	88.4	**87.9**
	SleePyCo (Lee et al., 2024)	86.2	80.1	81	90.6	47.3	88.8	87.4	86.6
	MultiChannelSleepNet (Dai et al., 2023)	86.5	81.6	80.3	92.6	47	89.5	88.3	83.8
	SeqSleepNet (Phan et al., 2019b)	85.2	79	79.6	–	–	–	–	–
	SleepEEGNet (Mousavi et al., 2019)	84.3	79	79.7	89.2	52.2	86.8	85.1	85
	DeepSleepNet (Supratak et al., 2017)	81.9	76	76.6	86.7	45.5	85.1	83.3	82.6
Sleep-EDF-78	MSDC-SSRNet	**86.2**	**81.2**	**81.7**	**94.7**	**55.9**	**87.8**	**84.9**	**85.2**
	SleePyCo (Lee et al., 2024)	84.6	79	79.1	93.5	50.4	86.5	80.5	84.2
	SeqSleepNet (Phan et al., 2019b)	82.6	76	76.4	92.2	47.8	84.9	77.2	79.9
	TinySleepNet (Supratak and Guo, 2020)	83.1	77.1	78.1	92.8	51	85.3	81.1	80.3
	SleepTransformer (Phan et al., 2022)	81.4	74.3	74.3	91.7	40.4	84.3	77.9	77.2
	AttnSleep (Eldele et al., 2021)	81.3	74	75.1	92	42	85	82.1	74.1
	SleepEEGNet (Mousavi et al., 2019)	80	73	73.6	91.7	44.1	82.5	73.5	76.1
	MultiChannelSleepNet (Dai et al., 2023)	84.9	78.9	79.4	94	52.8	86.3	81.5	82.6
SHHS	MSDC-SSRNet	**86.7**	**79.3**	**80.8**	**93**	**54.8**	**82.3**	**85.2**	**88.5**
	AttnSleep (Eldele et al., 2021)	84.2	78	75.3	86.7	33.2	87.1	87.1	82.1
	SeqSleepNet (Phan et al., 2019b)	86.5	81	78.5	–	–	–	–	–
CSPH	MSDC-SSRNet	**80.4**	**72.6**	**78.1**	**86.9**	**59.4**	**84.9**	**84.3**	**74.8**
	AttnSleep (Eldele et al., 2021)	79.4	71.6	77.6	86	60.7	84.3	82.5	74.2
	SleePyCo (Lee et al., 2024)	78.3	70.4	76.5	85.3	58.2	83.6	83.2	72.4
	MultiChannelSleepNet (Dai et al., 2023)	77.6	68.8	75.9	84.7	57.7	82.8	82.6	71.7
	SalientSleepNet (Liang et al., 2023)	77.3	68.9	76.5	84.4	60.1	82.5	83.5	72.1

Best metric values are marked in boldface.

To demonstrate the high accuracy of our method on the CSPH dataset, we compare it with four state-of-the-art methods, namely: (1) AttnSleep (Eldele et al., 2021); (2) SleepyPyCo (Lee et al., 2024); (3) MultiChannelSleepNet (Dai et al., 2023); (4) SalientSleepNet (Liang et al., 2023); in the CSPH dataset, the overall performance of MSDC-SSRNet also surpasses that of other networks. It performed well in both healthy subjects and OSA patients, demonstrating its robustness in handling complex datasets with varied sleep conditions. While SleePyCo (Lee et al., 2024) excels on simpler datasets such as Sleep-EDF-20 and Sleep-EDF-78, its performance declines when dealing with the more complex characteristics of the CSPH dataset. In addition, MSDC-SSRNet performs well in distinguishing the easily confused N2 and N3. Since there is a certain overlap in the transition period between the N2 and N3 stages, such as the overlapping delta waves (0.5–4 Hz) in the N3 stage and the sleep spindle waveform in the N2 stage, the distinction between the two is blurred. MSDC-SSRNet effectively helps doctors distinguish the N2 and N3 stages more accurately through auxiliary feature extraction and precise modeling.

Unlike SeqSleepNet (Phan et al., 2019b), which predicts the middle epoch using a recurrent architecture with three epochs as input, thereby slowing down the training process, the AttnSleep (Eldele et al., 2021) model adopts multi-scale feature extraction through varied convolutional kernel sizes and strides on the same input. In contrast, our MFEM utilizes dilated convolutions to enlarge the receptive field without significantly increasing the parameters, thereby enhancing local feature representation. This capability is crucial for sleep stage analysis, which requires detecting features at different time scales. Moreover, while AttnSleep (Eldele et al., 2021) shows improved F1-scores in certain stages like N2 and N3 compared to other models like SeqSleepNet (Phan et al., 2019b), it still falls short of MSDC-SSRNet in terms of overall accuracy and generalization across diverse datasets. MSDC-SSRNet reduces the heterogeneity between different modalities and data, proving to be a more versatile and efficient model in both accuracy and consistency.

### 3.5 Ablation experiments

As depicted in Table 5, we conducted ablation experiments on the Sleep-EDF-20 and CSPH dataset to assess the efficacy of various modules. Comparing BL, BL + CCTE, BL + MFEM, and our MSDC-SSRNet model reveals improvements across all metrics with each module's inclusion.

**Table 5:** Ablation experiment results for sleep-EDF-20 and CSPH datasets.

**Ablation experiment**	**Sleep-EDF-20 metrics**			**Sleep-EDF-20 Per-class F1 score**				
	**ACC**	**MF1**	**Kappa**	**W**	**N1**	**N2**	**N3**	**REM**
BL	86.5	80.3	81.6	92.9	47.0	89.5	88.3	83.8
BL + MFEM	86.8	81.5	82.0	93.1	51.1	89.6	88.7	84.8
BL + CCTE	87.9	82.0	83.4	93.9	50.9	90.2	89.1	86.0
MSDC-SSRNet	88.7	83.6	84.6	94.5	55.7	90.9	89.6	86.9
**Ablation experiment**	**CSPH metrics**			**CSPH Per-class F1 score**				
	**ACC**	**MF1**	**Kappa**	**W**	**N1**	**N2**	**N3**	**REM**
BL	75.3	74.5	68.1	82.4	53.6	80.7	80.3	70.5
BL + MFEM	77.5	76.0	68.8	84.7	58.6	82.8	82.7	71.8
BL + CCTE	79.5	77.3	71.2	85.8	57.7	83.9	83.5	73.6
MSDC-SSRNet	80.4	78.1	72.6	86.9	59.4	84.9	84.3	74.8

In the CSPH dataset, the CCTE module can significantly enhance classification performance, with overall improvements in ACC, MF1, and Kappa by 0.9%, 0.8% and 1.4%, respectively. F1 scores for each sleep stage also improved. We use the basic transformer as the baseline. Comparing it to the second variant, BL + MFE, we conclude that CCTE is essential for capturing frequent sleep stage transition features. However, MFEM is more effective in distinguishing the N1 stage, as the multi-scale feature extraction method allows the model to focus on finer features at lower or higher frequencies, thereby increasing overall sensitivity and reducing the impact of OSA on the model. In the Sleep-EDF-20 dataset, the final model shows an improvement in F1 scores of 8.6% for the N1 stage and 3.3% for the REM stage compared to the baseline (BL). According to the American Academy of Sleep Medicine (AASM) rules, especially in the N1 and REM sleep stages, the EEG features share similar low-amplitude, multi-frequency (LAMF) activities, making the features between these stages indistinct. Addressing this issue, our model framework can more effectively differentiate features of various sleep stages, particularly distinguishing between the N1 and REM stages.

### 3.6 Sensitivity analysis

**Figure 8:**
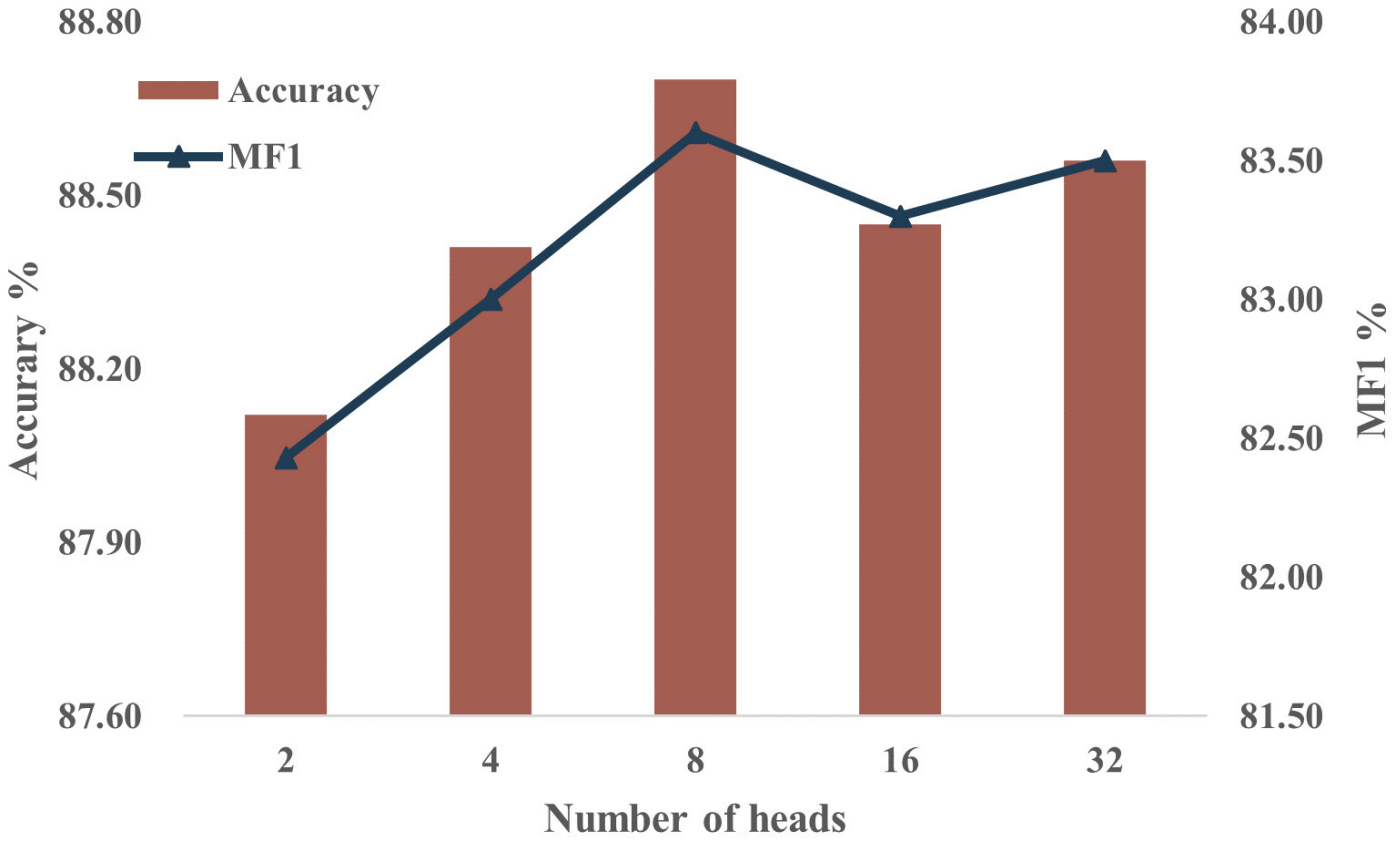
Performance on the sleep-EDF-20 dataset using different values of *H*.

Multi-head attention (MHA) is a pivotal element in our model, necessitating a sensitivity analysis regarding the number of heads employed. Given that the number of heads must be a divisor of the feature dimension *F* = 128, we set *H* to 2, 4, 8, 16, and 32 for the experiments, while maintaining constant values for the other parameters. Figure 8 shows the accuracy and MF1 scores of the model on the Sleep-EDF-20 dataset with different numbers of heads. The results show that model performance shows slight improvement with an increase in *H*. However, beyond a certain point, further increments in *H* lead to diminishing returns. This suggests that expanding the number of heads enhances feature capture initially, yet excessively dividing attention may reduce the per-head feature resolution. We select *H* = 8 as optimal for our model configuration based on these experimental findings.

In both the model's feature extraction and fusion processes, the CCTE encoder is utilized, so choosing an appropriate amount of encoders is also crucial. To further investigate the impact of the number of encoders *N*_*s*_ in single-channel feature extraction and *N*_*m*_ in multi-channel fusion, we keep other experimental parameters constant and use the Sleep-EDF-20 dataset. Initially, we fix *N*_*s*_ at 4, and repeat experiments with *N*_*m*_ values from {2, 4, 6, 8}, then fix *N*_*m*_ at 4, and repeat experiments with *N*_*s*_ values from {2, 4, 6, 8}. Based on the results shown in Table 6, changing the number of encoders does not significantly affect the model's overall performance. However, increasing *N*_*s*_ enhances the model's depth, improving its ability to capture features.

**Table 6:** Performance on the Sleep-EDF-20 dataset using different amounts of *N*_s_ and*N*_m_.

**Ns**	**Nm**	**Accuracy**	**MF1**
4	2	88.42	82.42
4	4	88.56	83.18
4	6	88.59	83.51
4	8	88.47	83.03
2	4	88.25	82.39
4	4	88.56	83.18
6	4	88.69	83.43
8	4	88.79	83.60

## 4 Discussion

MSDC-SSRNet uses multi-channel data for sleep staging tasks. Through ablation experiments and model stability analysis, each module in MSDC-SSRNet assists with sleep staging. The model performance is improved by capturing characteristic waves using multi-scale feature extraction and channel attention. While single-channel sleep staging algorithms are commonly used for portable home sleep monitoring, multi-channel data provides a more comprehensive view of sleep states. This comprehensive view aids the model in detecting subtler differences in sleep stages, which are more readily recognized by sleep physicians and offer better interpretability than single-channel systems.

As shown in Table 6, except for MultiChannelSleepNet (Dai et al., 2023), the staging performance of other single-channel models is inferior to MSDC-SSRNet. In a multi-channel framework, additional channels mitigate disruptions or poor signal quality in one channel, enhancing overall system robustness. In addition, the algorithm is applied to the self-built dataset CSPH. Unlike the public datasets, the subjects of this dataset suffer from OSA. The CSPH dataset is characterized by frequent sleep stage transitions and fragmented sleep cycles, which makes the sleep staging task challenging. Despite these difficulties, MSDC-SSRNet still performs well.

The CCTE captures long-range dependencies and enhances the importance of position information in the time-frequency domain. The MFEM uses different receptive fields to enhance the contribution of characteristic waves to sleep stages. The multi-scale attention layer integrates features with different weights, ensuring the preservation of multi-scale sleep transition rules. The model is able to characterize typical sleep stage features and distinguish them from other stages. EEG activity is highly dynamic, and multi-scale analysis can adapt to these changes, extracting significant features at different time scales to effectively capture short-term and long-term brain activity patterns. Compared to single-scale feature capture methods, the multi-scale approach provides a more stable feature representation, contributing to model generalizability and practical application.

Future research could address several limitations identified in this study. First, the data imbalance problem in the N1 stage still needs to be addressed. Additionally, our current model does not account for other relevant factors, such as age and gender, which could influence the study outcomes. Addressing these limitations in future research could further enhance the model's accuracy and applicability.

## 5 Conclusions

In this study, we introduced MSDC-SSRNet, a sleep staging model leveraging multi-scale dilated convolutions. It performs well on both healthy subjects and OSA subjects. In experiments with OSA subjects, the accuracy reaches 80.4%. This model utilizes the Channel-wise Convolutional Temporal Encoder (CCTE) and the Multi-Scale Feature Extraction Module (MFEM) for effective feature capture. The CCTE encoder employs a multi-head attention mechanism to capture long-range dependencies in the data. Additionally, we integrated CrossNorm, a novel normalization technique within CCTE, which enhances training data diversity by exchanging channel means and variances across feature maps. This ensures robust performance across diverse environmental and conditional data settings. The MFEM operates by capturing signals across a spectrum of frequencies from low to high, employing multi-scale feature extraction in the spatial domain. This module focuses on spatial feature extraction and adeptly captures various frequency components. This is particularly significant for EEG signals, as different frequency waveforms (such as δ, θ, α, β, and γ waves) exhibit distinct frequency characteristics.

Our model's effectiveness has been validated through comparisons with advanced models and extensive ablation experiments. Moreover, it provides more accurate predictions and classifications on datasets with specific clinical characteristics. Furthermore, we conducted a sensitivity analysis by varying the number of attention heads in the CCTE encoder for single-channel feature extraction and multi-channel fusion. This analysis demonstrated the model's stability and consistent performance under different parameter settings. The model's robust performance and adaptability to various configurations suggest its strong potential for real-world applications, particularly in clinical settings. Its high accuracy in classifying sleep stages for patients with obstructive sleep apnea makes it well-suited for deployment in home-based monitoring systems. Such systems could offer continuous, real-time sleep tracking, which would enhance patient convenience and accessibility while reducing the need for in-lab polysomnography. The model's ability to generalize across diverse patient populations further underscores its practical utility and potential for widespread implementation in both clinical and research environments.

## Data Availability

The datasets presented in this article are not readily available because protection of patient privacy, ensuring data security and ethical use. Requests to access the datasets should be directed to Jingxin Fan, fanjingxin2000@163.com.
